# Strongly Semicontinuous Domains and Semi-FS Domains

**DOI:** 10.1155/2014/262648

**Published:** 2014-08-17

**Authors:** Qingyu He, Luoshan Xu

**Affiliations:** Department of Mathematics, Yangzhou University, Yangzhou 225002, China

## Abstract

We are mainly concerned with some special kinds of semicontinuous domains and relationships between them. New concepts of strongly semicontinuous domains, meet semicontinuous domains and semi-FS domains are introduced. It is shown that a dcpo *L* is strongly semicontinuous if and only if *L* is semicontinuous and meet semicontinuous. It is proved that semi-FS domains are strongly semicontinuous. Some interpolation properties of semiway-below relations in (strongly) semicontinuous bc-domains are given. In terms of these properties, it is proved that strongly semicontinuous bc-domains, in particular strongly semicontinuous lattices, are all semi-FS domains.

## 1. Introduction

The theory of continuous lattices [1] and the more general theory of domains [2] initiated by Scott provide a mathematical foundation for the denotational semantics of programming languages and are closely linked to theoretical computer science, general topology and logics [3–5]. The purpose of the theory of domains is to give models for spaces to define computable functions. The idea is that the semantics of a programming language should be formally specified in terms of a small number of basic mathematical constructions on partial orders of information. Intuitively, we say that a state *x* approximates a state *y* if any computation of *y* yields the information of *x* at some finite stage. A logic-oriented approach to domain theory to formalize the properties of computation is provided in [2, 6].

So far, continuous lattices were generalized to other types of order structures, such as quasicontinuous posets [7, 8], *Z*-continuous posets [9], and *Z*-precontinuous posets [10]. Motivated by the concept of semiprime ideals studied by Rav in [11], Zhao in [12] first introduced the concept of semicontinuous lattices and showed that semicontinuous lattices have many properties similar to that of continuous lattices. Li and Wu [13] studied properties of semicontinuous lattices. Bi and Xu [14] introduced the semi-Scott topology and the semi-Lawson topology on semicontinuous lattices. Jiang and Shi [15] discussed characterizations of pseudoprimes and studied strong retracts of (stable) semicontinuous lattices. In [16], Li introduced semiprime sets and generalized semicontinuous lattices to semicontinuous domains.

Note that, in the definition of a semicontinuous lattice *L*, the condition used is that, for every element *x* ∈ *L*, ∨⇓*x* ≥ *x*. This condition is too weak to guarantee that every element can be approximated by elements which are below and semiway-below it in a semicontinuous lattice. To guarantee the mentioned approximation property in a suitable class of semicontinuous lattices, or even of semicontinuous domains, we in this paper introduce the concept of strongly semicontinuous domains. It turns out that strongly semicontinuous domains have domain-like features that every element can be approximated by elements below and semiway-below it. The concept of meet semicontinuous domains is also introduced. It is obtained that a dcpo is strongly semicontinuous if and only if it is semicontinuous and meet semicontinuous. Moreover, inspired by the definition of FS-domains posed by Jung [17] in the realm of domains, we introduce the concept of semi-FS domains which are defined by the existence of some approximating identities consisting of finitely separated functions in the realm of dcpos. It is proved that every semi-FS domain is a strongly semicontinuous domain and that every strongly semicontinuous bc-domain, in particular every strongly semicontinuous lattice, is a semi-FS domain. A counterexample is constructed to show that a strongly semicontinuous domain need not be a semi-FS domain.

We organize the paper as follows: Section 2 gives preliminaries; Section 3 investigates strongly semicontinuous domains; Section 4 introduces semi-FS domains and discusses their properties.

## 2. Preliminaries

We give some basic concepts and results which will be used in the sequel. Most of them come from [4, 12]. For other unstated concepts please refer to [16].

A subset *D* of a poset *L* is called* directed* (resp.,* filtered*) if it is nonempty and every finite subset of *D* has an upper (resp., a lower) bound in *D*. A poset in which every directed set has a supremum is called a* dcpo*. For a subset *A* of a poset *L*, let ↓*A* = {*y* ∈ *L*: ∃*x* ∈ *A*, *y* ≤ *x*} and ↑*A* = {*y* ∈ *L*: ∃*x* ∈ *A*, *y* ≥ *x*}. We say that *A* is a* lower set* (resp., an* upper set*) if ↓*A* = *A* (resp., ↑*A* = *A*). A subset of *L* is an* ideal* (resp., a* filter*) if and only if it is a directed lower set (resp., filtered upper set). A* principal ideal* (resp.,* principal filter*) is a set of the form ↓*x* = {*y* ∈ *L*: *y* ≤ *x*} (resp., ↑*x* = {*y* ∈ *L*: *x* ≤ *y*}). The set of all ideals (resp. filters) in *L* is denoted by *Id*(*L*) (resp., Filt(*L*)). For an ideal *P* ∈ *Id*(*L*), *P* is said to be* prime* if *L*∖*P* = *∅* or *L*∖*P* ∈ Filt(*L*). We denote the family of all prime ideals of *L* by *PI*(*L*). A poset is called* bounded complete*, if every subset that is bounded above has a supremum. A bounded complete dcpo is called a* bc-dcpo*.

Let *S*⊆*L*. We denote *S* ↓ = {*y* ∈ *L* : ∀*x* ∈ *S*, *y* ≤ *x*} and *S*↑ = {*y* ∈ *L* : ∀*x* ∈ *S*, *y* ≥ *x*} the set of upper bounds and lower bounds of *S*, respectively.

**Lemma 1 (see [16, Lemma 3.2.8]).**

Let *L* be a poset, *A*, *B*⊆*L*. If *A*⊆*B*, then we have

(1) $$\begin{array}{c} A↑↓\subseteq B↑↓,\;\;A↓↑\subseteq B↓↑. \end{array}$$

**Definition 2 (see [11]).**

Let *L* be a lattice. An ideal *I* of *L* is said to be* semiprime* if for any *x*, *y*, *z* ∈ *L*, *x*∧*y* ∈ *I* and *x*∧*z* ∈ *I* imply *x*∧(*y*∨*z*) ∈ *I*.

**Lemma 3 (see [12, Lemma 1.2]).**

An ideal *P* of a lattice *L* is semiprime if and only if there exist prime ideals *P*_*i*_  (*i* ∈ *I*) of *L* such that *P* = ⋂_*i*∈*I*_ *P*_*i*_.

Thus every prime ideal is semiprime, and *PI*(*L*)⊆*Rd*(*L*). And by Lemma 3, we can generalize the concept of semiprime ideals to the setting of posets.

**Definition 4 .**

Let *L* be a poset and *S*⊆*L* an ideal of *L*. If there is a family of prime ideals {*P*_*i*_}_*i*∈*I*_ such that *S* = ⋂_*i*∈*I*_ *P*_*i*_, then *S* is called a* semiprime ideal*. The family of all semiprime ideals of *L* is denoted by *Rd*(*L*).

Li in [16] generalized semiprime ideals to semiprime sets.

**Definition 5 (see [16]).**

Let *L* be a poset. A set *S*⊆*L* is said to be semiprime if there exists a family of prime ideals *P*_*i*_  (*i* ∈ *I*) of *L* such that *S* = ⋂_*i*∈*I*_ *P*_*i*_.

We denote the family of all semiprime sets of *L* with *SP*(*L*). Clearly, a semiprime set need not to be directed. If a semiprime set *S* is directed, then *S* is a semiprime ideal. For a dcpo, we have the following relation:

(2) $$\begin{array}{c} PI\left(L\right)\subseteq Rd\left(L\right)=SP\left(L\right)\cap Idl\left(L\right)\subseteq SP\left(L\right). \end{array}$$

**Proposition 6 .**

Let *L* be a bc-dcpo. Then *SP*(*L*) = *Rd*(*L*).

**Proof**

It suffices to show that *SP*(*L*)⊆*Rd*(*L*). Let *A* ∈ *SP*(*L*). Then by Definition 5 there exists a family of prime ideals *P*_*i*_  (*i* ∈ *I*) of *L* such that *A* = ⋂_*i*∈*I*_ *P*_*i*_. For any *y*_1_, *y*_2_ ∈ *A*, we have *y*_1_, *y*_2_ ∈ *P*_*i*_ for all *i* ∈ *I*. Since *P*_*i*_ is directed, there exists *y* ∈ *P*_*i*_ such that *y*_1_, *y*_2_ ≤ *y*. As *L* is a bc-dcpo, we see that *y*_1_∨*y*_2_ exists and *y*_1_∨*y*_2_ ∈ *P*_*i*_ for all *i* ∈ *I*. Therefore, *y*_1_∨*y*_2_ ∈ *A*. So, *A* is directed and *A* ∈ *Rd*(*L*), as desired.

**Lemma 7 .**

For a bc-dcpo *L*, let *M*⊆*L* and *L* ^*⊤*^ = *L* ∪ {*⊤*} be the complete lattice obtained from *L* by adjoining a top element *⊤*. Then we have

- *M* ∈ *PI*(*L*) if and only if *M* ∈ *PI*(*L* ^*⊤*^);
- *M* ∈ *Rd*(*L*) if and only if *M* ∈ *Rd*(*L* ^*⊤*^).

**Proof**

(i) Suppose that *M* ∈ *PI*(*L* ^*⊤*^). Since *M*⊆*L*, we have *L*∖*M* = *L* ^*⊤*^∖*M* ∈ Filt(*L*) and *M* ∈ *PI*(*L*).

Conversely, let *M* ∈ *PI*(*L*). Then *M* ∈ *Id*(*L*) and *L*∖*M* ∈ Filt(*L*). So, *L* ^*⊤*^∖*M* = (*L*∖*M*)∪{*⊤*} ∈ Filt(*L* ^*⊤*^) and *M* ∈ *PI*(*L* ^*⊤*^).

(ii) Suppose that *M* ∈ *Rd*(*L*). Then by Definition 4, *M* = ⋂_*i*∈*I*_ *P*_*i*_, where *P*_*i*_ ∈ *PI*(*L*)  (*i* ∈ *I*). It follows from (i) above that *P*_*i*_ ∈ *PI*(*L* ^*⊤*^) for all *i* ∈ *I*; thus, *M* ∈ *Rd*(*L* ^*⊤*^).

Conversely, let *M* ∈ *Rd*(*L* ^*⊤*^). By Lemma 3, there exist prime ideals *P*_*i*_  (*i* ∈ *I*) of *L* ^*⊤*^ such that *M* = ⋂_*i*∈*I*_ *P*_*i*_. Since *⊤* ∉ *M*, *⊤* ∉ *P*_*i*_0__ for some *i*_0_ ∈ *I*. Let *J* = {*j* ∈ *I* : *⊤* ∉ *P*_*j*_}. Then for any *i* ∈ *I*∖*J*, we see that *⊤* ∈ *P*_*i*_ = *L* ^*⊤*^. By (i) above, we have *P*_*j*_ ∈ *PI*(*L*) for each *j* ∈ *J*. Then *M* = ⋂_*j*∈*J*_ *P*_*j*_, and *M* ∈ *SP*(*L*). By Proposition 6, *M* ∈ *Rd*(*L*).

In a poset *L*, we say that *x* is* way-below y*, or *x approximates y*, written *x* ≪ *y*, and if *D* is directed with existing sup⁡*D* and sup⁡*D* ≥ *y*, then *x* ≤ *d* for some *d* ∈ *D*. Equivalently, *x* ≪ *y* iff *x* ∈ *I* for every ideal *I* of *L* such that *y* ≤ sup⁡*I* whenever sup⁡*I* exists. We use ↡*x* to denote the set {*a* ∈ *L* : *a* ≪ *x*}. If *L* is a dcpo and, for every element *x* ∈ *L*, the set ↡*x* is directed and sup⁡↡*x* = *x*, then *L* is called a* domain*. A domain *L* is called an* L-domain* if for each *x* ∈ *L*, the principal ideal ↓*x* is a complete lattice. A complete lattice which is a domain is called a* continuous lattice*.

For complete lattices, replacing ideals with semiprime ideals, Zhao in [12] defined a weak form of the way-below relation.

**Definition 8 (see [12]).**

Let *L* be a complete lattice. Define the semiway-below relation ⇐ on *L* as follows: for *x*, *y* ∈ *L*, *x* ⇐ *y* if for any semiprime ideal *I* of *L*, *y* ≤ ∨*I* implies *x* ∈ *I*. For each *x* ∈ *L*, we write ⇓*x* = {*y* ∈ *L* : *y* ⇐ *x*}, ⇑*x* = {*y* ∈ *L* : *x* ⇐ *y*}.

**Definition 9 (see [12]).**

A complete lattice *L* is said to be* semicontinuous*, if for any *x* ∈ *L*, *x* ≤ ∨(⇓*x*).

Zhao [12] showed that the interpolation property holds in semicontinuous lattices.

**Theorem 10 (see [12, Theorem 1.8]).**

If *L* is a semicontinuous lattice, then *x* ⇐ *y* implies the existence of a *z* ∈ *L* such that *x* ⇐ *z* ⇐ *y*.

## 3. Strongly Semicontinuous Domains

In terms of semiprime sets, semicontinuous lattices can be generalized to semicontinuous domains. And then strongly semicontinuous domains will be defined.

**Definition 11 .**

Let *L* be a poset. Define the relation ⇐ on *L* as follows: for any *x*, *y* ∈ *L*, *y* ⇐ *x* if for any semiprime set *S* of *L*, *x* ∈ *S*↑↓ implies *y* ∈ *S*. An element *k* of *L* is said to be* semicompact* if *k* ⇐ *k*. For each *x* ∈ *L*, we write ⇓*x* = {*y* ∈ *L* : *y* ⇐ *x*} and ⇑*x* = {*y* ∈ *L* : *x* ⇐ *y*}.

**Proposition 12 .**

Let *L* be a dcpo; then,

(3) ⇓x=∩{S∈SP(L):x∈S↑↓}=∩{S∈Rd(L):x≤∨S}=∩{P∈PI(L):x≤∨P}.

So, for each *x* ∈ *L*, ⇓*x* ∈ *SP*(*L*). If *L* is a bc-dcpo, then ⇓*x* ∈ *Rd*(*L*).

**Proof**

Note that *J*↑↓ = ↓(∨*J*) for each *J* ∈ *Id*(*L*). So, by Lemma 1, we have that

(4) ⇓x =∩{S∈SP(L):x∈S↑↓}⊆∩{S∈Rd(L):x∈↓(∨S)} ⊆∩{P∈PI(L):x∈↓(∨P)}.

Let *A* = ∩{*P* ∈ *PI*(*L*) : *x* ∈ ↓(∨*P*)}. Then *A* ∈ *SP*(*L*).

Next we show that *A*⊆⇓*x*. Let *y* ∈ *A*. For any *M* ∈ *SP*(*L*) with *x* ∈ *M*↑↓, by Definition 5 there exists a family of prime ideals {*P*_*i*_}_*i*∈*I*_ such that *M* = ⋂_*i*∈*I*_ *P*_*i*_. It follows from Lemma 1 that *x* ∈ *M*↑↓⊆*P*_*i*_↑↓ = ↓(∨*P*_*i*_) for each *i* ∈ *I*. Since *y* ∈ *A* = ∩{*P* ∈ *PI*(*L*) : *x* ∈ ↓(∨*P*)}, we have that *y* ∈ *P*_*i*_ for each *i* ∈ *I* and *y* ∈ ⋂_*i*∈*I*_ *P*_*i*_ = *M*. By Definition 11, *y* ⇐ *x*. So, *A*⊆⇓*x*, as desired.

In [16], the semiway-below relation ⇐_*s*_ on a poset was defined [16, Definition 3.2.2] in a different way. For a dcpo *L* and *x* ∈ *L*, it is established in [16, Proposition 3.2.5] that ⇓_*s*_ *x* = ∩{*P* ∈ *PI*(*L*) : *x* ≤ ∨*P*}. So, by Proposition 12 above, we see that ⇓*x* = ⇓_*s*_ *x*. This means that for a dcpo *L* and *x*, *y* ∈ *L*, *y* ⇐_*s*_ *x*⇔*y* ⇐ *x*. Thus, in the setting of dcpos, Definition 11 is equivalent to Definition 3.2.2 in [16].

Note that for a poset *L* and *x*, *y* ∈ *L*, *y* ⇐_*s*_ *x* need not imply *y* ⇐ *x*.

**Remark 13 .**

Let *L* be a dcpo and *a*, *b*, *c*, *d* ∈ *L*. Then it is easy to check that

1. *a* ⇐ *b* does not imply *a* ≤ *b*, the typical modular lattice *M*_5_ is a counterexample;
2. if *a* ≤ *b* ⇐ *c* ≤ *d*, then *a* ⇐ *d*. If *a* ⇐ *b* ⇐ *c*, then *a* ⇐ *c*;
3. if *a*∨*b* exists and *a*, *b* ⇐ *c*, then *a*∨*b* ⇐ *c*;
4. for any *a*, *b* ∈ *L*, *a* ≪ *b* implies *a* ⇐ *b*.

**Definition 14 (see [16]).**

A dcpo *L* is called a* semicontinuous dcpo* if for all *x* ∈ *L*, *x* ∈ (⇓*x*)↑↓. A semicontinuous dcpo will also be called a* semicontinuous domain*. A bc-dcpo which is semicontinuous will be called a* semicontinuous bc-domain*.

By Proposition 12, one can immediately have the following.

**Proposition 15 .**

Let *L* be a dcpo. Then *L* is semicontinuous iff for any *x* ∈ *L*, ⇓*x* is the smallest semiprime set *S* such that *x* ∈ *S*↑↓.

**Proposition 16 .**

Let *L* be a dcpo. If, for any *x* ∈ *L*, there exists a subset *A*⊆⇓*x* and *x* ∈ *A*↑↓, then *L* is semicontinuous.

**Proof**

It follows from Lemma 1 and Definition 14.

Strengthening the condition in Definition 14, we give the following.

**Definition 17 .**

A dcpo *L* is said to be* strongly semicontinuous* if for each *x* ∈ *L*,

(5) $$\begin{array}{c} x\in \left(⇓x\cap ↓x\right)↑↓. \end{array}$$

A dcpo (bc-dcpo) which is strongly semicontinuous will be called a* strongly semicontinuous domain* (*strongly semicontinuous bc-domain*). A complete lattice which is strongly semicontinuous will be called a* strongly semicontinuous lattice*.

Clearly, every strongly semicontinuous domain is semicontinuous, a semicontinuous domain *L* satisfying the condition *x* ∈ (⇓*x*∩↓*x*)↑↓(∀*x* ∈ *L*) is strongly semicontinuous. It is easy to see that, for a dcpo *L* without proper prime ideals, every pair of elements in *L* has the semiway-below relation ⇐ and *L* is strongly semicontinuous. However, a semicontinuous domain need not be strongly semicontinuous. The following counterexample first appeared in [18].

**Example 18 (see [18]).**

Let *L* = {⊥, *a*, *b*, *x*, *⊤*}∪{*x*_*n*_ : *n* = 0,1,…}. The partial order on *L* is defined by ⊥ ≤*a*, *b* ≤ *x*_0_ ≤ ⋯≤*x*_*n*_ ⋯ ≤*⊤*, ⊥ ≤*x* ≤ *⊤*. It is clear that the prime ideals of *L* are *L*∖↑*x* and *L*. We observe that, for any *t* ∈ *L*, ⇓*t* = *L*∖↑*x* and *t* ≤ ∨⇓*t*. Thus, *L* is a semicontinuous domain. However, note that, in this example *x* ∉ (⇓*x*∩↓*x*)↑↓, which yields that *L* is not strongly semicontinuous.

**Proposition 19 .**

Every domain is a strongly semicontinuous domain.

**Proof**

It follows from Remark 13 (4) that ∀*x* ∈ *L*, ↡*x*⊆⇓*x*∩↓*x*. By Lemma 1, *x* ∈ ↓*x* = ↓(∨↡*x*) = (↡*x*)↑↓⊆(⇓*x*∩↓*x*)↑↓. Thus, *L* is a strongly semicontinuous.

Wu and Li in [19] introduced the following concept of meet semicontinuous lattices.

**Definition 20 (see [19]).**

A complete lattice *L* is said to be* meet semicontinuous* if for any *x* ∈ *L* and *I* ∈ *Rd*(*L*), *x*∧(∨*I*) = ∨(*x*∧*I*).

It is known that a semicontinuous lattice need not be a meet semicontinuous lattice. However, it is proved in [18] that strongly semicontinuous lattices are all meet semicontinuous. Generalize meet semicontinuous lattices, we have

**Definition 21 .**

A dcpo *L* is said to be* meet semicontinuous* if for any *x* ∈ *L* and *S* ∈ *SP*(*L*), (↓*x*∩*S*)↑↓ = ↓*x*∩*S*↑↓.

It is easy to check that, for complete lattices, the meet semicontinuity in Definitions 20 and 21 are equivalent.

**Proposition 22 .**

Every strongly semicontinuous domain is meet semicontinuous.

**Proof**

Suppose that *L* is a strongly semicontinuous domain. For any *x* ∈ *L* and *S* ∈ *SP*(*L*), by Lemma 1, it is easy to see that ↓*x*∩*S*↑↓⊇(↓*x*∩*S*)↑↓. So, it suffices to show that ↓*x*∩*S*↑↓⊆(↓*x*∩*S*)↑↓. To this end, let *t* ∈ ↓*x*∩*S*↑↓. Then *t* ∈ *S*↑↓ and *t* ∈ (⇓*t*∩↓*t*)↑↓ since *L* is strongly semicontinuous. For any *r* ∈ ⇓*t*∩↓*t*, we have that *r* ⇐ *t* ∈ *S*↑↓ and *r* ≤ *t* ≤ *x*. Thus, by Definition 11, *r* ∈ *S* and *r* ∈ ↓*x*∩*S*. By the arbitrariness of *r* ∈ ⇓*t*∩↓*t*, we see that ⇓*t*∩↓*t*⊆↓*x*∩*S*. Therefore, *t* ∈ (⇓*t*∩↓*t*)↑↓⊆(↓*x*∩*S*)↑↓. So, ↓*x*∩*S*↑↓⊆(↓*x*∩*S*)↑↓, as desired.

**Remark 23 .**

Note that, for the complete lattice in Example 18 and the prime ideal *L*∖↑*x*, we have ↓*x*∩(*L*∖↑*x*)↑↓≠(↓*x*∩(*L*∖↑*x*))↑↓, revealing that *L* is not meet semicontinuous.

**Theorem 24 .**

A dcpo *L* is strongly semicontinuous iff *L* is semicontinuous and meet semicontinuous.

**Proof**

⇒: By Proposition 22.

⇐: For each *x* ∈ *L*, by the semicontinuity of *L* we have that *x* ∈ (⇓*x*)↑↓. It follows from meet semicontinuity of *L* that *x* ∈ ↓*x*∩(⇓*x*)↑↓ = (↓*x*∩⇓*x*)↑↓. So, by Definition 17, *L* is strongly semicontinuous.

**Proposition 25 .**

For a (strongly) semicontinuous bc-domain *L*, let *L* ^*⊤*^ = *L* ∪ {*⊤*} be the complete lattice obtained from *L* by adjoining a top element *⊤*. Then *L* ^*⊤*^ is a (strongly) semicontinuous lattice.

**Proof**

Firstly, we show that ⇓_*L*^*⊤*^_ *x*⊇⇓_*L*_ *x* for each *x* ∈ *L*. Let *y* ⇐_*L*_ *x*. Then for any *P* ∈ *Rd*(*L* ^*⊤*^) with ∨_*L*^*⊤*^_ *P* ≥ *x*, if *P* = *L* ^*⊤*^, then *y* ∈ *P*. If *P* ≠ *L* ^*⊤*^, then *⊤* ∉ *P* and *P*⊆*L*. By Lemma 7 (ii), *P* ∈ *Rd*(*L*). Thus, ∨_*L*_ *P* = ∨_*L*^*⊤*^_ *P* ≥ *x* and *y* ∈ *P*. So, *y* ⇐_*L*^*⊤*^_ *x* and ⇓_*L*_ *x*⊆⇓_*L*^*⊤*^_ *x* for each *x* ∈ *L*.

If *L* itself is a (strongly) semicontinuous lattice, then *⊤* is isolated in *L* ^*⊤*^ and *L* ^*⊤*^ is trivially a (strongly) semicontinuous lattice.

For a bc-dcpo *L* without the biggest element, we see that ∨_*L*^*⊤*^_ *L* = *⊤*. So, if *L* is semicontinuous, then ∨_*L*^*⊤*^_⇓_*L*^*⊤*^_ *x* ≥ ∨_*L*^*⊤*^_⇓_*L*_ *x* ≥ *x* for each *x* ∈ *L*. It follows from

(6) $$\begin{array}{c} {⇓}_{L^{⊤}}⊤⊇\cup_{x\in L}{⇓}_{L^{⊤}}x⊇\cup_{x\in L}{⇓}_{L}x \end{array}$$

that ∨_*L*^*⊤*^_(⇓_*L*^*⊤*^_ *⊤*∩↓*⊤*) = ∨_*L*^*⊤*^_⇓_*L*^*⊤*^_ *⊤* ≥ ∨_*L*^*⊤*^_∪_*x*∈*L*_⇓_*L*_ *x* ≥ ∨_*L*^*⊤*^_ *L* = *⊤*. If *L* is strongly semicontinuous, then for all *x* ∈ *L*, *x* ≥ ∨_*L*^*⊤*^_(⇓_*L*^*⊤*^_ *x*∩↓⁡*x*) ≥ ∨_*L*^*⊤*^_(⇓_*L*_ *x*∩↓*x*) ≥ *x* and ∨_*L*^*⊤*^_(⇓_*L*^*⊤*^_ *x*∩↓*x*) = *x*.

To sum up, if *L* is a semicontinuous bc-domain, then ∀*y* ∈ *L* ^*⊤*^, *y* ≤ ∨_*L*^*⊤*^_⇓_*L*^*⊤*^_ *y* and *L* ^*⊤*^ is a semicontinuous lattice; if *L* is a strongly semicontinuous bc-domain, then ∀*y* ∈ *L* ^*⊤*^, ∨_*L*^*⊤*^_(⇓_*L*^*⊤*^_ *y*∩↓*y*) = *y*, and *L* ^*⊤*^ is a strongly semicontinuous lattice.

**Corollary 26 .**

If *L* is semicontinuous bc-domain, then ∀*x* ∈ *L*, ⇓_*L*^*⊤*^_ *x* = ⇓_*L*_ *x*.

**Proof**

By the proof of Proposition 25, we have that ⇓_*L*^*⊤*^_ *x*⊇⇓_*L*_ *x* and *L* ^*⊤*^ is a semicontinuous lattice. Then it follows from Propositions 12 and 15 and Lemma 7 (ii) that ⇓_*L*^*⊤*^_ *x*⊆⇓_*L*_ *x*. So, ⇓_*L*^*⊤*^_ *x* = ⇓_*L*_ *x*.

Whether the interpolation property holds or not in semicontinuous domains is still unknown. So, by Theorem 10 and Corollary 26, we immediately have the following.

**Corollary 27 .**

If *L* is a (strongly) semicontinuous bc-domain, then *x* ⇐ *y* implies the existence of a *z* ∈ *L* such that *x* ⇐ *z* ⇐ *y*.

Next we will show that (strongly) semicontinuous bc-domains also exhibit some strong types of interpolation properties.

**Proposition 28 .**

In a (strongly) semicontinuous bc-domain *L*,

(i) for all *x*, *y* ∈ *L* with *x* ≤ *y*, one has
  (7) $$\begin{array}{c} \left({SI}^{\leq }\right)\;\;x⇐y\;implies\;\left(\exists z\in L\right)\;\left(x⇐z⇐y,\;\;x\leq z\right); \end{array}$$
(ii) for all *x*, *y* ∈ *L* with *x* < *y*, one has
  (8) $$\begin{array}{c} \left({SI}^{<}\right)\;\;x⇐y\;implies\;\left(\exists z\in L\right)\;\left(x⇐z⇐y,\;\;x<z\right) \end{array}$$

**Proof**

(i) For any *x*, *y* ∈ *L*, *x* ≤ *y*, and *x* ⇐ *y*, it follows from Corollary 27 that there exists a *z*_0_ ∈ *L* such that *x* ⇐ *z*_0_ ⇐ *y*. Noticing that ⇓*y* ∈ *Rd*(*L*), by Proposition 6, there exists a *w* ∈ *Rd*(*L*) such that *x*, *z*_0_ ≤ *w*. Since *L* is a bc-dcpo, *x*∨*z*_0_ exists and *x* ⇐ *z*_0_ ≤ *x*∨*z*_0_ ⇐ *y*. Thus, (*SI* ^≤^) holds.

(ii) Let *x*, *y* ∈ *L* with *x* < *y*, and *x* ⇐ *y*. By Corollary 27 there exists a *z*_0_ ∈ *L* such that *x* ⇐ *z*_0_ ⇐ *y*. Since ⇓*y* ∈ *Rd*(*L*) and *y* ≤ ∨⇓*y*, there exists *z*_1_ ∈ ⇓*y* such that *z*_1_≰*x*. Noticing that *z*_0_, *z*_1_ ∈ ⇓*y* ∈ *Rd*(*L*), we see that *z*_0_∨*z*_1_ exists. Set *z* = *z*_0_∨*z*_1_; then, *x* < *z*. Therefore, (*SI* ^<^) holds.

## 4. Semi-FS Domains

In this section, we introduce semi-FS domains which are counterparts of FS-domains posed by Jung [17] in the setting of strongly semicontinuous domains. It is proved that every strongly semicontinuous bc-domain, in particular every strongly semicontinuous lattice, is a semi-FS domain.

**Definition 29 (see [16]).**

Let *L*, *M* be dcpos. A function *f* : *L* → *M* is said to* preserve suprema of prime ideals* if it is order-preserving and for any *P* ∈ *PI*(*L*), *f*(⋁*P*) = ⋁*f*(*P*).

Let *L*, *M* be dcpos. We use [*L*, *M*] to denote all order-preserving functions from *L* to *M*, use [*L*↪*M*] to denote all the functions preserving suprema of prime ideals from *L* to *M* and use [*L* → *M*] to denote all the Scott-continuous functions from *L* to *M*. All of them are under the pointwise order. It is easy to see that [*L* → *M*]⊆[*L*↪*M*]⊆[*L*, *M*].

**Proposition 30 .**

Let *L*, *M* be dcpos. Then [*L*↪*M*] is a dcpo.

**Proof**

Let *G* be a directed subset of [*L*↪*M*] and *f*(*x*) = ∨_*g*∈*G*_ *g*(*x*) for all *x* ∈ *L*. Then it is easy to see that *f* is order-preserving. For any prime ideal *P* ∈ *PI*(*L*), we have

(9) ∨f(P)=∨x∈Pf(x)=∨x∈P∨g∈Gg(x)=∨g∈G∨g(P)=∨g∈Gg(∨P)=f(∨P).

Thus, *f* ∈ [*L*↪*M*], showing that [*L*↪*M*] is a dcpo.

**Definition 31 .**

Let *L* be a dcpo. If *D*⊆[*L*, *L*] is directed and sup⁡_*δ*∈*D*_ *δ* = *Id*_*L*_, then we say that *D* is an* approximate identity* for *L*.

**Proposition 32 .**

Let *L* be a dcpo. If *L* has an approximate identity *D*⊆[*L*↪*L*] such that *δ*(*x*) ⇐ *x* for all *δ* ∈ *D* and for all *x* ∈ *L*, then *L* is a strongly semicontinuous domain.

**Proof**

For any *x* ∈ *L*, let *A* = {*δ*(*x*) : *δ* ∈ *D*}. Then *A*⊆⇓*x* ∩↓*x*. Note that *D* is directed and *x* ∈ ↓(∨*A*) = *A*↑↓. By the Proposition 16 and Definition 17, it follows that *L* is a strongly semicontinuous domain.

**Definition 33 (see [17]).**

Let *L* be a dcpo. A function *δ* : *L* → *L* on *L* is finitely separating if there is a finite set *F*_*δ*_ such that, for each *x* ∈ *L*, there exists *y* ∈ *F*_*δ*_ such that *δ*(*x*) ≤ *y* ≤ *x*.

A dcpo *L* is called* a semi-FS domain* if there is an approximate identity *D*⊆[*L*↪*L*] consisting of finitely separating functions.

For an FS-domain *L*, there is an approximate identity *D*⊆[*L* → *L*]⊆[*L*↪*L*] for *L* consisting of finitely separating functions. So, an FS-domain is a semi-FS domain.

**Proposition 34 .**

Let *L* be a dcpo. If *δ* ∈ [*L*↪*L*] is finitely separating, then, for all *x* ∈ *L*, *δ*(*x*) ⇐ *x*. Thus a semi-FS domain is a strongly semicontinuous domain.

**Proof**

Suppose that *x* ∈ *L* and *P* ∈ *PI*(*L*) with *x* ≤ ∨*P*. Since *δ* is finitely separating, there exists a finite set *F*_*δ*_ such that for each *d* ∈ *P* there exists *y*_*d*_ ∈ *F*_*δ*_ with *δ*(*d*) ≤ *y*_*d*_ ≤ *d*. Let *F*_*δ*_′ = {*y*_*d*_ ∈ *F*_*δ*_ : *d* ∈ *P*}, a nonempty finite subset of *F*_*δ*_. Then for each *y* ∈ *F*_*δ*_′, we can get *d*_*y*_ ∈ *P* such that *δ*(*d*_*y*_) ≤ *y* ≤ *d*_*y*_. As *P* is a prime ideal, there exists *d*_0_ ∈ *P* such that *y* ≤ *d*_*y*_ ≤ *d*_0_ for all *y* ∈ *F*_*δ*_′. Hence for all *d* ∈ *P*, *δ*(*d*) ≤ *y*_*d*_ ≤ *d*_0_ and *δ*(*x*) ≤ *δ*(∨*P*) = ∨_*d*∈*P*_ *δ*(*d*) ≤ *d*_0_. Therefore, *δ*(*x*) ∈ *P*. It follows from Proposition 12 that *δ*(*x*) ⇐ *x*.

By Proposition 32 we see that *L* is a strongly semicontinuous domain.

The next example gives a strongly semicontinuous domain which is not a semi-FS domain, showing that the reverse of Proposition 34 is not true.

**Example 35 .**

**Figure 1:**
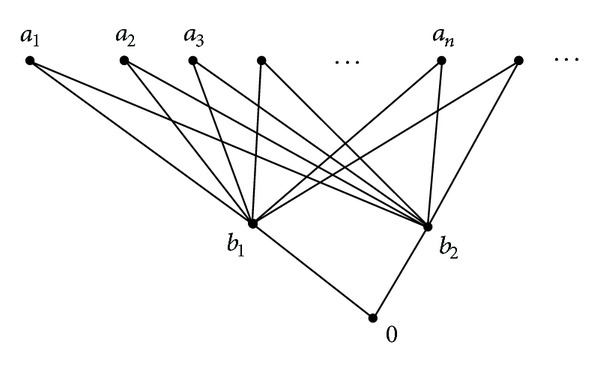
*L*  is strongly semicontinuous but not a semi-FS domain.

Let *L* = {0, *b*_1_, *b*_2_}∪{*a*_*j*_ : *j* = 1,2,…} be the domain showing in Figure 1, where 0 < *b*_*i*_ < *a*_*j*_ for *i* = 1,2 and *j* = 1,2,…. It is clear that *L* is an *L*-domain but not compact in the Lawson topology. By [20, Corollary 2.2], we immediately see that *L* is not an FS-domain. Note that, in *L*, every directed set is finite. So, an order-preserving function from *L* to *L* preserves directed sups in *L*. And [*L* → *L*]⊆[*L*↪*L*]⊆[*L*, *L*] = [*L* → *L*]. So, [*L* → *L*] = [*L*↪*L*]. Since *L* is not an FS-domain, *L* is not a semi-FS domain either.

**Proposition 36 .**

Every strongly semicontinuous bc-domain is a semi-FS domain.

**Proof**

Let *L* be a strongly semicontinuous bc-domain. For each *x* ∈ *L*, *S* ∈ *P*_fin_(*L*), define *δ*_*S*_ : *L* → *L* by *δ*_*S*_(*x*) = ∨{*y* ∈ *S*∩↓*x* : *y* ⇐ *x*}. If {*y* ∈ *S*∩↓*x* : *y* ⇐ *x*} = *∅*, then *δ*_*S*_(*x*) = ⊥, the least element of *L*. So, *δ*_*S*_(*x*) is well-defined. It is easy to see that *δ*_*S*_ is order-preserving with *δ*_*S*_(*x*) ≤ *x* for all *x* ∈ *L*. Next we show that *δ*_*S*_ preserves suprema of prime ideals. For each *P* ∈ *PI*(*L*), it suffices to show that *δ*_*S*_(∨*P*) = *δ*_*S*_(*k*) ≤ ∨_*p*∈*P*_ *δ*_*S*_(*p*), where *k* = ∨*P*. If {*y* ∈ *S*∩↓*k* : *y* ⇐ *k*} = *∅*, then, by the definition of *δ*_*S*_, we see that *δ*_*S*_(*k*) = ⊥ ≤∨_*p*∈*P*_ *δ*_*S*_(*p*). Let {*y*_1_,…, *y*_*l*_} = {*y* ∈ *S*∩↓*k* : *y* ⇐ *k*} ≠ *∅*, then *m* = ∨_*i*=1_^*l*^ *y*_*i*_ exists in *L*. By the definition of *δ*_*S*_, *δ*_*S*_(*k*) = *m* ≤ *k*. Since *y*_*i*_ ⇐ *k* for *i* = 1,2,…, *l*, we have that *m* ⇐ *k*. By (SI^≤^) in Proposition 28, there is a m* ≥ *m* such that *m* ⇐ m* ⇐ *k*. It follows from m* ⇐ *k* = ∨*P* that *m*, m* ∈ *P*. Noticing that *m* ≤ m* and *y*_*i*_ ≤ *m* ⇐ m* ∈ *P*, we have {*y*_1_,…, *y*_*l*_}⊆{*y* ∈ *S*∩↓m* : *y* ⇐ m*} which yields that ∨_*p*∈*P*_ *δ*_*S*_(*p*) ≥ *δ*_*S*_(m*) ≥ *δ*_*S*_(*k*). So, *δ*_*S*_ preserves suprema of prime ideals, and *δ* ∈ [*L*↪*L*].

Suppose that *S*, *T* ∈ *P*_fin_(*L*) with *S*⊆*T*. It is easy to see that *δ*_*S*_ ≤ *δ*_*T*_ and *D* = {*δ*_*S*_}_*S*∈*P*_fin_(*L*)_ is directed. Let *F*_*δ*_*S*__ = {*δ*_*S*_(*x*) : *x* ∈ *L*}. Then *F*_*δ*_*S*__ is finite by the finiteness of *S*. So, *δ*_*S*_ is a finitely separating function. Since *L* is strongly semicontinuous, for each *x* ∈ *L* and *δ*_*S*_ ∈ *D*, *δ*_*S*_(*x*) = ∨{*y* ∈ *S*∩↓*x* : *y* ⇐ *x*} ≤ *x* and

(10) ∨δS∈DδS(x)=∨δS∈D(∨{y∈S∩↓x:y⇐x})≥∨z⇐x(∨{y∈{z}∩↓x:y⇐x})=∨(⇓x∩↓x)=x.

Therefore, *D* = {*δ*_*S*_}_*S*∈*P*_fin_(*L*)_⊆[*L*↪*L*] is an approximate identity for *L* consisting of finitely separating functions, and *L* is a semi-FS domain.

**Corollary 37 .**

Every strongly semicontinuous lattice is a semi-FS domain.
